# Occupational exposure to particles and biomarkers of cardiovascular disease—during work and after vacation

**DOI:** 10.1007/s00420-022-01900-5

**Published:** 2022-07-11

**Authors:** Karin Grahn, Karin Broberg, Per Gustavsson, Petter Ljungman, Petra Lindfors, Mattias Sjöström, Pernilla Wiebert, Jenny Selander

**Affiliations:** 1 Institute of Environmental Medicine, Karolinska Institutet, Stockholm, Sweden; 2 Centre for Occupational and Environmental Medicine, Region Stockholm, Stockholm, Sweden; 3 Division of Occupational and Environmental Medicine, Lund University, Lund, Sweden; 4 Department of Cardiology, Danderyd University Hospital, Danderyd, Sweden; 5 Department of Psychology, Stockholm University, Stockholm, Sweden

**Keywords:** Cardiovascular risk markers, Occupational exposure, Particles, Longitudinal measures, Construction industry, Respirable silica

## Abstract

**Objective:**

Ambient particle matter is a risk factor for cardiovascular disease (CVD). However, little is known about associations between particles in occupational settings and risk of CVD. We investigated associations between occupational dust exposure and biomarkers of CVD, and potential recovery effects after vacation.

**Methods:**

Personal dust exposure measurements (respirable silica, respirable dust < 4 µm, and particles of 0.1–10 µm (PM 0.1–10) were conducted once, and biological sampling were performed twice on non-smoking, male construction workers in Stockholm county, Sweden; during work and immediately after summer vacation. Linear regressions with adjustments for confounders and covariates were performed evaluating associations between occupational dust exposure and biomarkers. Paired *t* tests were performed evaluating changes before and after vacation.

**Results:**

Sixty-five workers participated. Homocysteine concentrations were significantly higher with increasing concentrations (mg/m^3^) of respirable silica, respirable dust, and PM 0.1–10, and pulse rate with higher levels of respirable dust and dust of PM 0.1–10. Homocysteine levels were also positively correlated to number of years of dust exposure, as were low-density lipoprotein (LDL) levels. A clear recovery effect was present for LDL after vacation, but not for homocysteine.

**Conclusions:**

Occupational dust exposure was associated with some CVD risk markers, even at mean exposure concentrations below the Swedish occupational exposure limits for respirable silica and respirable dust, respectively. Vacation resulted in recovery for some risk markers. However, the change of the homocysteine and LDL levels suggest a long-term effect. Reduction of occupational exposure to dust may decrease the risk of CVD among exposed workers.

**Supplementary Information:**

The online version contains supplementary material available at 10.1007/s00420-022-01900-5.

## Introduction

Cardiovascular disease (CVD) is a leading cause of death in the world, with approximately 18 million lives lost each year (WHO 2020). CVD includes all diseases of the heart and vessels typically also affecting other organ systems like the brain, kidneys, lungs and peripheral tissue (WHO 2019). Risk factors include non-modifiable factors like sex, age (Rodgers et al. 2019) and family history of CVD (Leander et al. 2001; Lloyd-Jones et al. 2004) as well as modifiable risk factors, such as lifestyle factors (Stewart et al. 2017) and occupational risks (Kristensen 1989a, 1989b). Identifying all types of risk factors, particularly modifiable factors from lifestyle and occupational settings, is crucial for effectively preventing CVD.

Particulate matter in ambient air is a risk factor both for development and aggravation of CVD, (Leander et al. 2019; Polichetti et al. 2009). There is strong evidence in support for a causal relationship between exposure to ambient fine particulate matter < 2.5 µm (PM 2.5) and adverse cardiovascular effects (Brook et al. 2010; Leander et al. 2019). However, less is known about the associations between particulate matter in occupational settings and CVDs, even though exposure levels in occupational settings often are considerably higher than in residential areas. There are several dust-exposed occupations with increased risks of CVD, for example miners (Chen et al. 2012; Weiner et al. 2007), agricultural workers (Sjogren et al. 2003), chimney sweeps (Gustavsson et al. 2013) with ischemic heart disease, and manual workers with stroke (Sjogren et al. 2013). A systematic review of observational studies on occupational exposure to particles and CVD concluded that particle exposure may be associated with mortality from ischemic heart disease (IHD) and non-fatal myocardial infarction (MI), as well as with decreased heart rate variability and systemic inflammation (Fang et al. 2010). Also, studies on traffic enforcers in the metro system suggest an association between low-level exposure to cadmium-dust and ventricular arrythmias (Regencia et al. 2022). However, causality remains to be determined due to potential uncontrolled confounding in the available studies. We also lack a clear understanding regarding the dose–response relationships of different occupational particle exposures and CVD (SBU 2017).

Mainly, three mechanistic pathways have been suggested for the associations between inhalation of particulate matter and adverse cardiovascular effects (Franklin et al. 2015). The first is a “spill-over” of mediators of oxidative stress and inflammation that are generated in the lungs into the systemic circulation. This systemic inflammatory response may release for example acute phase proteins such as C-reactive protein (CRP), serum amyloid A (SAA), and coagulation marker fibrinogen (Arant et al. 2009; Franklin et al. 2015). Negative effects include for example vascular oxidative stress, atherosclerosis, or dysfunction of the endothelium. The second pathway is an imbalance in the autonomic nervous system. This leads to (more) short-term effects such as rapid changes in blood pressure or heart rate but may also lead to long-term effects including development of diseases such as hypertension (Franklin et al. 2015). The third pathway involves the translocation of certain particles into the circulation which affects cardiovascular tissues negatively (Franklin et al. 2015). Particle composition, their chemical characteristics, and sizes may activate different pathways (Brook et al. 2010).

The inflammatory markers CRP, SAA and coagulation marker fibrinogen are all established biomarkers of cardiovascular disease (Danesh et al. 1998; Danesh et al. 2005; Danesh et al. 2000). Other markers commonly used to indicate risk of CVD include lipid biomarkers cholesterol, low-density lipoprotein (LDL), high-density lipoprotein (HDL), and triglycerides (Gordon et al. 1977; Kannel et al 1961; Lewington et al. 2007) as well as the one-carbon metabolism biomarker homocysteine (Eikelboom et al. 1999; Wald et al. 2002).

The aim of this study was to investigate if occupational exposure to particles is associated with CVD risk markers. Furthermore, the study also investigates if there is a recovery effect after vacation.

## Material and methods

### Study design and study participants

To study exposure to respirable silica as well as respirable dust and dust of other sizes in the occupational setting, active construction workers were selected as the study population. Workplaces and work tasks with different levels of exposure to dust, including respirable silica, were identified in collaboration with representatives of the Construction Trade Union (Svenska byggnadsarbetareförbundet), the Association for Concrete/Stone drillers, Demolition and Decontamination Workers (Branschföreningen för byggnadsberedning) and large construction companies. Based on this information, occupations among construction workers were classified into high- and low-exposure to respirable dust, and study participants were recruited to ensure a high contrast in exposure levels. The high-exposed group consisted of “concrete and demolition workers”, including concrete/stone drillers, demolition workers, concrete workers, plasterers and bricklayers. The low-exposed group “carpenters” included construction carpenters and plumbers. We restricted the study to men who had not smoked for the past 6 months at the first measurements at work, since sex and smoking are established risk factors for CVD that can vary with exposure. The cut-off of 6 months was chosen to enable enough time for recovery effects of recent smoking on the markers of cardiovascular health and to avoid too few study participants since smoking is rather common in this work sector. Furthermore, participants were to have had worked in the construction industry for at least 6 months to enable the analysis of dose–response relationships of chronic effects.

The workers were recruited during late Autumn 2018 and Spring 2019, in collaboration with the above-mentioned association, trade union and large construction companies. In total, 28 construction companies in Stockholm County were contacted. Contact persons were asked to invite their employees or subcontractors to participate in the study.

Each contact person and study participant received overall information about the study, an informed consent form and a questionnaire. After the form and questionnaire had been completed, personal measurements of exposure were performed and biological samples were collected on the worksites. Sampling was performed on Tuesdays to Thursdays when participants had worked at least one day after the weekend and worked for a full day.

### Biological sampling and exposure measurements

Exposure measurements were conducted once, and biological sampling twice for each participant as follows: the first occasion (measurements during work) took place at the participants’ worksite during Spring (March–June 2019), and the second occasion (measurements after vacation) took place at the Clinic of Occupational and Environmental Medicine, Stockholm County, directly after the participants’ summer vacation (July–September 2019) and before returning to work.

### Biological sampling

Biological sampling during work took place in the morning, before the participants started their working day. Height and weight were measured, and samples of peripheral blood (total volume 50 ml) were collected. After at least 5 min of rest, blood pressure and pulse rate were measured 3 times with 1–2 min in between when the participants were sitting down (ABPM pulse rate monitor, model ABPM 50, GIMA S.p.A., Italy). Biological sampling after vacation was performed in the same way as during work and took place at the clinic in the morning.

### Analyses of biological samples

Peripheral whole blood samples were taken, and after 15 min serum and plasma were separated by centrifugation for 10 min (fibrinogen for 20 min) and stored at − 20 °C. In the end of the same week (mostly Thursdays), samples were frozen at − 80 °C. Serum and plasma samples were used to analyse (level of detection within parentheses) CRP (0.3 mg/L), homocysteine (3 µmol/L), cholesterol (0.1 mmol/L), HDL (0.08 mmol/L), LDL (0.1 mmol/L), triglycerides (0.1 mmol/L), SAA (0.8 mg/L) and fibrinogen (0.3 g/L), by routine methods at the Department of Clinical Chemistry, Lund University Hospital. All samples were analysed in December 2019 (all but SAA) and February 2020 (SAA) in the same batch and randomised. Thus, the samples collected at measurements during work were stored for 6–9 months (all but SAA) and 8–11 months (SAA), and the samples collected at the measurements at clinic were stored fo 3–5 months (all but SAA) and 5–7 months (SAA).

### Exposure measurements

Each participant had their average physical workload for the whole working day assessed by an occupational hygienist (Arbetsmiljöverket 2011). In addition, participants recorded their main work tasks every 15 min in a diary. Respirable silica and respirable dust concentrations (filter measurements) and dust of 0.1—10 µm (PM 0.1–10) concentrations (continuously logged data), were measured during one working day at each participant’s worksite. The filters (25 mm membrane) with preseperator aluminium cyclone (SKC Inc, USA) were placed at the participant’s shoulder, close to the breathing zone. Respirable silica and respirable dust were collected on the filters using AirChek XR5000 sample pumps with a flow of 2.5 L/min. Airflow was measured using ChekMate flow meter model 375–07,550 (SKC Inc, USA). The device DataRAM pDR-1000AN Monitor for logging dust concentrations was placed on the participant’s back. DataRAM pDR-1000AN Monitor measured dust of PM 0.1–10-concentrations, which covers an air concentration range of 0.001–400 mg/m^3^ (size range 0.1–10 µm) using pDR-COM software version 2.10 (ThermoFisher Scientific, USA).

### Analyses of exposure measurements

Respirable silica and respirable dust collected on membrane filters were analysed at the accredited Analysis Laboratory, University Hospital, Örebro, Sweden, using gravimetric and x-ray diffraction analysis to determine the mass of respirable dust and respirable silica, respectively, in mg (respirable dust: SS-EN 481 1993, respirable silica: SS-ISO 16258-2:2015 Arbetsplatsluft—Bestämning av kristallin kiseldioxid med röntgendiffraktion,). The level of detection (LOD) for respirable silica was 0.002 mg/sample, and for respirable dust 0.10 mg/sample. Samples with levels below LOD were assigned the value 0 mg/m^3^ in the analyses. Then sampled air volume was considered, and the results were presented as mg/m^3^.

The average concentration of dust of PM 0.1–10 in mg/m^3^ during the workday was obtained from the DataRAM instrument for each subject. This dust encompasses all types of particles in the air, not only respirable silica and respirable dust.

For every participant, we recorded the use of respirator masks and hearing protectors. When a mask or hearing protector was used, the type (filter or air-supplied mask, earmuffs or ear plugs, respectively) as well as duration of use was noted.

Each participant's work period in years with exposure above background level was determined in consensus by two hygienists, based on information given in the questionnaires.

### Questionnaire

There were two different questionnaires, one administered during work and the other after vacation. During work, the questionnaire asked about age (years), ever smoking (yes/no), ever smoking (years of smoking), alcohol consumption (< = 4 or > 4 times per week), diet (vegetables < 5 or >  = 5 times per week), and exercise habits (< 1 or >  = 1 time per week of 30 min regular physical activity). Additionally, the questionnaire covered current exposure to noise (yes/no), whole-body vibration (yes/no), hand-arm vibration (yes/no), working in cold temperature (yes/no), diesel fumes (yes/no), chemical vapours/gases (yes/no), welding fumes (yes/no), dust other than silica (yes/no)and physically demanding work (yes/no), as well as years in profession (years). Moreover, the questionnaire included psychosocial work characteristics such as mentally demanding work (yes/no), as well as questions of exposure to respirable silica from hobbies (yes/no). Other variables evaluated were prior CVD—including myocardial infarction (MI), angina pectoris, hypertension, stroke, thrombosis in arm/leg, other heart disease—(yes/no), kidney/urinary disease, diabetes—(yes/no), family history of MI (yes/no), family history of stroke (yes/no), family history of hypertension (yes/no), prescribed medicine use (yes/no), and non-prescribed medicine use (yes/no).

After the vacation, participants completed another short questionnaire about vacation activities involving exposure to silica, health status including prescribed medicine use and tobacco use. The prescribed medication was further classified as CVD-related or not by a cardiologist (Petter Ljungman). The questionnaires from both sampling occasions included the question “Do you have an ongoing infection, for example a common cold, inflammation, or flu?”.

### Statistical analyses

To evaluate differences in characteristics between carpenters’ and concrete and demolition workers’ groups and between exposure categories’ 75th percentile concentration levels of respirable silica, respirable dust and dust of PM 0.1–10 groups, two-sample *t* tests were performed for continuous variables and Fischer’s exact tests were performed for categorical variables. Linear regression was performed evaluating the associations between continuous variables of the exposures (1) respirable silica, (2) respirable dust, and (3) dust of PM 0.1–10, and the outcomes blood pressure, resting pulse and biomarkers of CVD. Sensitivity analysis with adjustments for age, body mass index (BMI), common viral infections, CVD-related drugs at measurements during work, alcohol consumption and physical activity was also performed. Also, linear regression was performed to evaluate associations between dichotomized variables according to the 50th and 75th percentiles (the low group as reference) of respirable silica, respirable dust and dust of PM 0.1–10, respectively, and the risk markers.

According to the 75th percentile dichotomized exposure groups (low and high) of respirable silica, respirable dust and dust of PM 0.1–10, respectively, paired *t* tests were performed to evaluate the change in blood pressure, resting pulse and biomarkers of cardiovascular disease before and after vacation. Sensitivity analysis on subjects having no common viral infection, not medicating with any CVD-related drug at any of the two sampling occasions, or not having worked prior to vacation measurement, was also performed.

Association between duration of dust exposure (working years) above background level, crude and adjusted for BMI, and BMI and age (years), respectively, and blood pressure, resting pulse and biomarkers was also calculated using linear regression with continuous variables.

Correlations between different dusts were calculated using Spearman rank tests.

Statistical analyses were performed with StataCorp LLC STATA/SE version 16.1.

## Results

The response rate for participation in the study for the companies was 71% (20 out of 28). The response rate among participants remains unknown since recruitments were administered by the managers or a person responsible for the work environment at each company.

In total, 65 male non-smoking construction workers were included as follows: 29 were concrete and demolition workers (ten concrete/stone drillers, ten demolition workers, six concrete workers, two plasterers and one bricklayer), and 36 were carpenters (35 construction carpenters and one plumber). Results are presented based on the measured levels of exposure to the different dusts. Table 1 therefore, shows individual and occupational risk factor characteristics of all study participants based on the exposure to respirable silica, respirable dustand dust of PM 0.1–10, respectively, stratified on the 75th percentile. Workers with exposure below ( <) and equal to or above (≥) the 75th percentile did not differ significantly, except for alcohol consumption and physical activity in the respirable dust analyses where the high-exposed group (≥ 75th percentile) reported less alcohol consumption (*p* = 0.03) and more physical activity (*p* = 0.04). Furthermore, workers high-exposed to respirable silica were younger (*p* = 0.01) and reported having worked for less years in dusty professions (*p* = 0.02) compared to the low-exposed, and more workers high-exposed to dust of PM 0.1–10 were observed during the day of measurement to have used respiratory masks (*p* = 0.01) compared to the low- exposed. Albeit non-significant, participants in the high-exposed respirable dust and dust of PM 0.1–10 groups were younger and had a lower BMI than those in the low-exposed groups. The high-exposed groups also worked less years in their profession. The groups high-exposed to respirable dust and dust of PM 0.1–10 had a higher proportion of ever smokers, but the groups high- and low-exposed to respirable silica had similar proportions of ever smokers, although all high-exposed groups had smoked longer. There were no significant differences in characteristics between the two occupational groups, apart from use of mask (*p* < 0.00) and exposure to whole body vibration (*p* = 0.05), where the concrete and demolition workers reported a higher frequency (Tables S1, S2).

**Table 1:** Characteristics of study participants stratified on exposure measurements’ 75th percentile of respirable silica concentration (0.026 mg/m^3^), 75th percentile of respirable dust concentration (0.462 mg/m^3^ and 75th percentile of dust of PM 0.1–10 concentration (0.984 mg/m^3^)

	Respirable silica						Respirable dust						Dust of PM 0.1–10					
	< 75th percentile *n* = 49			≥ 75th percentile *n* = 16			< 75th percentile *n* = 49			≥ 75th percentile *n* = 16			< 75th percentile *n* = 38			≥ 75th percentile *n* = 13		
	Mean	Min–max	*n* (%)	Mean	Max	*n* (%)	Mean	Min–max	*n* (%)	Mean	Max	*n* (%)	Mean	Min–max	*n* (%)	Mean	Max	*n* (%)
**Individual risk factors**																		
Age (years)	42	20–65		32.7	21–55		41	20–65		35	21–57		39.6	20–65		35.2	21–57	
BMI (kg/m^2^)^a^	29	19–46		26.4	20–35		29	19–46		26	20–35		28.0	19–41		25.3	20–34	
Ever smoker (yes)^b^			19 (37)			7 (38)			19 (40)			7 (47)			13 (35)			7 (54)
Ever smoking (years)	10	3–25		15.3	1–26		10	3–25		15	1–26		9.8	3–25		10.8	1–20	
Alcohol consumption^c^			11 (23)			1 (6)			12 (25)			0 (0)			7 (19)			1 (8)
Vegetable consumption^d^			35 (73)			9 (56)			36 (75)			8 (50)			26 (70)			6 (46)
Physical activity (high)^e^			20 (41)			10 (67)			19 (39)			11 (73)			20(53)			7 (58)
**Occupational risk factors**^f^																		
Years in dusty profession	16	0–43		8	0–39		15	0–43		10	0–39		14	0–43		11.0	1–39	
Respiratory mask use^g^			7 (15)			4 (25)			7 (15)			4 (25)			4 (11)			6 (46)
Noise			48 (100)			16 (100)			48 (100)			16 (100)			37 (100)			13 (100)
Hearing protector use^h^			46 (94)			15 (94)			47 (96)			14 (88)			37 (97)			11 (85)
Vibration whole-body			20 (41)			11 (69)			22 (45)			9 (56)			16 (42)			8 (62)
Vibration hand/arm			46 (96)			15 (100)			46 (96)			15 (100)			36 (100)			13 (100)
Working in cold temperature			42 (86)			16 (100)			43 (88)			15 (94)			35 (92)			11 (85)
Diesel fumes			20 (41)			9 (56)			23 (47)			6 (38)			20 (53)			5 (38)
Chemical vapours/gases			17 (35)			6 (40)			18 (38)			5(33)			16 (43)			4 (31)
Welding fumes			16 (33)			5 (33)			17 (35)			4 (25)			14 (37)			3 (25)
Dust other than silica			42 (89)			14 (93)			41 (89)			15 (94)			32 (89)			12 (100)
Physically demanding work			46 (96)			16 (100)			47 (96)			15 (100)			38 (100)			12 (100)
Mentally demanding work			26 (58)			12 (80)			27 (60)			11 (73)			24 (71)			9 (69)
Hobby exposure to RCS^i^			8 (16)			4 (27)			9 (18)			3 (20)			7 (18)			4 (33)

Percent (%) calculated from complete responses

^a^Body mass index, calculated from height and weight measurements

^b^Respirable silica and Respirable dust: including 4 participants who are party-smokers (3 in the group < 75th percentile, 1 in the group ≥ 75th percentile) and Dust of PM 0.1–10: including 3 participants who are party-smokers (in the group < 75th percentile)

^c^>  = 4 times/week

^d^>  = 5 times/week

^e^Once a week or more of minimum 30 min regular physical activity

^f^Yes for all variables except Years in dusty profession

^g^Observed usage by occupational hygienist during the day of measurement. Type of respirator mask: filter or air-supplied

^h^Observed usage by occupational hygienist during the day of measurement. Type of hearing protection: ear muffs or ear plugs

^i^Respirable crystalline silica

**Fig. 1:**
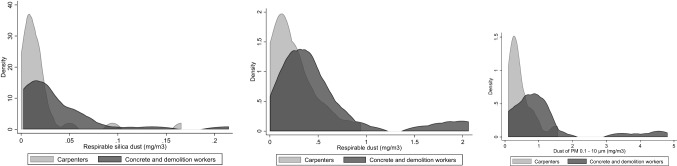
Kernel density of the personal sampling exposure concentration levels (mg/m^3^) of respirable silica, respirable dust, and dust of PM 0.1–10. Dichotomized based on occupational groups of construction workers: “carpenters” (red in figure) and “concrete and demolition workers” (grey in figure)

Figure 1 shows personal sampling exposure levels (mg/m^3^) of respirable silica, respirable dust, and dust of PM 0.1–10 for carpenters as well as concrete and demolition workers. The mean levels of respirable silica (0.037 mg/m^3^) and respirable dust (0.510 mg/m^3^) in the concrete and demolition workers’ group were approximately twice the levels among carpenters’ group (0.019 and 0.228 mg/m^3^, respectively), and the mean level of dust of PM 0.1–10 among concrete and demolition workers (1.185 mg/m^3^) was approximately 2.5 times the level among carpenters (0.461 mg/m^3^). However, the exposure distributions were skewed to the right and overlapped to a high degree between the occupational groups. Also, a small number of concrete and demolition workers with very high exposures had a strong influence on the mean concentrations in this group. The Swedish occupational exposure limits (OEL) are 0.1 mg/m^3^ for respirable silica, and 2.5 mg/m^3^ for respirable inorganic dust, respectively (Arbetsmiljöverket 2018). None of the occupational groups exhibited such mean levels although at individual level, three workers, two concrete and demolition workers and one carpenter, had mean levels that were higher during their 8-h working day.

Exposure to respirable silica and respirable dust was highly correlated (*r*_S_ = 0.83) as were respirable dust and dust of PM 0.1–10 (*r*_S_ = 0.82). Exposure to respirable silica and dust of PM 0.1–10 was moderately correlated (*r*_S_ = 0.60). The median of the proportions of respirable silica to respirable dust in general were 6.8% for concrete and demolition workers and 6.2% for carpenters.

Table 2 presents linear regression estimates per 1 mg/m^3^ increments of the associations between concentrations of respirable silica, respirable dust and dust of PM 0.1–10 and cardiovascular biomarkers, adjusted for age and BMI, for all workers. Homocysteine concentrations correlated positively and significantly with all three dust types. Exposure to respirable dust and dust of PM 0.1–10 was also significantly associated negatively with blood concentrations of HDL and positively with resting pulse rate.

**Table 2:** Effect estimates expressed as *β* values with 95% confidence intervals (CI) evaluated by linear regression analysis of all workers and adjusted for age and body mass index

	Respirable silica continuous variables		Respirable dust continuous variables		Dust of PM 0.1–10 continuous variables	
	Adj *β*^a^	95% CI	Adj *β*^a^	95% CI	Adj *β*^a^	95% CI
Systolic blood pressure (mm Hg)	− 11.93	− 100.63, 76.77	0.88	− 7.37, 9.14	1.78	− 1.96, 5.51
Diastolic blood pressure (mm Hg)	− 13.01	− 84.68, 58.65	0.98	− 5.69, 7.65	1.31	− 1.37, 3.99
C-reactive protein (mg/L)	0.74	− 17.81, 19.29	0.68	− 1.04, 2.39	0.23	− 0.54, 1.01
Homocystein (µmol/L)^b^	25.22	2.97, 47.48	2.24	0.16, 4.33	1.20	0.22, 2.18
Cholesterol (mmol/L)	− 1.96	− 7.99, 4.07	− 0.27	− 0.83, 0.29	− 0.11	− 0.37, 0.15
High density lipoprotein (mmol/L)	− 1.11	− 3.20, 0.99	− 0.22	− 0.41, − 0.03	− 0.09	− 0.19, − 0.00
Low density lipoprotein (mmol/L)	− 0.82	− 6.01, 4.37	− 0.18	− 0.66, 0.30	− 0.05	− 0.27, 0.18
Triglycerides (mmol/L)	− 1.53	− 10.03, 6.96	0.00	− 0.79, 0.80	− 0.01	− 0.43, 0.40
Pulse, resting (bpm)	28.77	− 38.70, 96.24	8.09	2.13, 14.06	3.77	1.07, 6.46
Serum amyloid A (mg/L)	− 1.38	− 21.60, 18.83	− 0.10	− 1.98, 1.78	0.10	− 0.73, 0.92
Fibrinogen (g/L)	1.60	− 1.76, 4.97	0.15	− 0.17, 0.46	0.07	− 0.09, 0.23

The *β* represents the change in each variable with an increase in exposure of 1 mg/m^3^

^a^Linear regression, adjusted for age and body mass index (BMI)

^b^1 missing

Since high alcohol consumption and physical activity differed between the workers stratified on the 75th percentile concentrations of respirable dust, we performed sensitivity analyses adjusting for these potential confounders for all three types of dust. These analyses showed small differences from Table 2, (Table S3). For statistical power reasons the smaller model adjusting for only age and BMI was chosen for the main analyses in this manuscript. We also performed analyses for respirable silica, respirable dust and dust of PM 0.1–10 using linear regression on dichotomized groups (50th and 75th percentile) with the low group used as reference. The results showed similar patterns, see Supplementary material, Table S4. Six workers reported having a common viral infection at the first biological sampling during work, and seven workers used CVD-related drugs. However, sensitivity analyses adjusting for infections and CVD-related drugs showed similar results, see Supplementary material, Table S5. Also, sensitivity analyses adjusting for age, BMI and storage time showed similar point estimates as in Table 2 but with wider confidence intervals, hence indicating no degrading of samples, data not shown.

Table 3 presents the difference between measurements during work and after vacation for blood pressure, resting pulse and the biomarkers. Among workers with concentrations ≥ 75th percentile of respirable silica, respirable dust and dust of PM 0.1–10, systolic blood pressure was significantly higher during work than after vacation, with the largest mean change (4.87 mm Hg) for respirable dust exposure. The diastolic blood pressure was significantly higher during work for participants high-exposed to respirable dust (mean change 3.98 mm Hg). Among participants high-exposed to respirable silica and respirable dust, LDL-concentrations were significantly higher during work, mean change 1.27 and 1.54 mmol/L, respectively. Furthermore, CRP-concentrations were significantly lower during work among study participants high-exposed to respirable silica and dust of PM 0.1–10, mean change 0.83 and 1.37 mmol/L, respectively. There were no significant changes of homocysteine-concentrations after vacation.

**Table 3:** Change in blood pressure, pulse, and biomarkers between measurements during work and after vacation (before and after vacation)

	75th percentile (0.026 mg/m^3^) of respirable silica				75th percentile (0.462 mg/m^3^) of respirable dust				75th percentile (0.984 mg/m^3^) of dust of PM 0.1–10			
	Low, *n* = 49		High, *n* = 16		Low, *n* = 49		High, *n* = 16		Low, *n* = 38		High, *n* = 13	
	Mean of change^b^	*p*^a^	Mean of change^c^	*p*^a^	Mean of change^d^	*p*^a^	Mean of change^e^	*p*^a^	Mean of change^e^	*p*^a^	Mean of change^d^	*p*^a^
Systolic blood pressure (mm Hg)	0.57	0.68	3.75	0.01	0.31	0.82	4.87	< 0.01	0.11	0.95	3.88	< 0.01
Diastolic blood pressure (mm Hg)	0.11	0.93	2.58	0.17	− 0.25	0.84	3.98	0.05	− 0.52	0.71	1.97	0.29
C-reactive protein (mg/L)	− 0.43	0.28	− 0.83	0.03	− 0.39	0.30	− 0.99	0.06	− 0.83	0.05	− 1.37	0.02
Homocysteine (µmol/L)	− 0.07	0.89	− 0.33	0.63	− 0.23	0.60	0.21	0.80	− 0.42	0.48	0.09	0.92
Cholesterol (mmol/L)	− 0.04	0.63	− 0.26	0.07	− 0.12	0.18	− 0.03	0.87	− 0.09	0.32	0.01	0.97
High density lipoprotein (mmol/L)	− 0.03	0.43	− 0.02	0.46	− 0.03	0.33	− 0.01	0.84	− 0.02	0.52	0.00	0.98
Low density lipoprotein (mmol/L)	1.29	< 0.01	1.27	< 0.01	1.21	< 0.01	1.54	< 0.01	1.47	< 0.01	0.59	0.53
Triglycerides (mmol/L)	− 0.14	0.34	− 0.09	0.47	− 0.15	0.26	− 0.02	0.89	− 0.06	0.59	− 0.37	0.41
Pulse, resting (bpm)	− 3.18	0.05	1.49	0.63	− 4.02	0.01	4.69	0.08	− 4.82	0.01	0.69	0.86
SerumAmyloid A (mg/L)	− 1.35	0.16	− 1.78	0.13	− 1.15	0.21	− 2.48	0.12	− 2.09	0.07	− 3.41	0.06
Fibrinogen (g/L)	− 0.01	0.87	− 0.08	0.62	− 0.01	0.86	− 0.08	0.68	− 0.10	0.24	− 0.09	0.68

Dichotomized (low and high exposure) based on exposure measurements’ 75th percentile concentrations of respirable silica, respirable dust, and dust of PM 0.1–10, respectively. A positive value indicates a higher value during work as compared to after vacation

^a^Paired *t* test

^b^2 missing for all markers except for homocysteine, and high density lipoprotein with 3 missing

^c^1 missing for all markers except for fibrinogen with 2 missing

^d^1 missing for all markers except for homocysteine, and high density lipoprotein with 2 missing

^e^2 missing for all markers except for fibrinogen with 3 missing

In all workers exposed to concentrations < 75th percentile of respirable silica, respirable dust and dust of PM 0.1–10 no significant changes were seen in blood pressure, but LDL-concentrations were significantly higher and resting pulse lower during work than after vacation.

Nine workers had a common viral infection at any of the two measurements, and 12 workers had worked prior to the second measurement. Sensitivity analyses on participants without CVD- medication, and participants free from infections at the two biological sampling occasions, or not having worked prior to measurements after vacation, showed similar results, see Supplementary material, Table S6a–c.

Table S7 presents linear regression analyses of number of years of occupational exposure to dust. When adjusting for both BMI and age, the homocysteine- and LDL-concentrations were positively and significantly correlated to number of years in dust-exposed jobs. The homocysteine levels were 0.11 (95% CI 0.02–0.21) µmol/L and LDL levels 0.03 (95% CI 0.01–0.05) mmol/L higher, respectively, for each additional working year.

## Discussion

In this study of low-to-moderate occupational exposure to particulate matter, we found that higher exposure to particulate matter during work was associated with CVD risk markers, with recovery for some markers, but not all, during vacation. More specifically, higher concentrations of all types of dust assessed during work were significantly associated with higher concentrations of homocysteine. Higher levels of respirable dust and dust of PM 0.1–10 were significantly associated with a higher resting pulse and significantly lower HDL concentrations. After recovery during vacation, a significant decrease of LDL-concentrations was found for most workers, and among workers with the higher concentrations of dust (≥ 75th percentile) a significant decrease of systolic blood pressure was found.

Elevated concentrations of homocysteine have been linked to pathogenetic processes of CVD such as oxidative stress, impaired endothelial function, induction of thrombosis, as well as atherosclerosis and hypertension (Eikelboom et al. 1999; Haynes 2002; Refsum et al. 2004; Wald et al. 2002). Within our study, the homocysteine concentrations increased with higher concentrations of all three types of dusts, and the number of years of occupational dust exposure was also significantly associated with higher homocysteine concentrations. There was no clear recovery effect after vacation. Taken together, this indicates that exposure to dust may be associated with a long-lasting upregulation of homocysteine concentrations. Physical activity (Silva and da Mota 2014) and alcohol consumption (Sakuta and Suzuki 2005) could possibly affect the homocysteine levels, but sensitivity analyses on all three types of dust adjusting for these two potential confounders resulted in small effects on the association between dust and homocysteine concentrations (Table S3).

This study’s finding that occupational dust exposure is associated with elevated concentrations of homocysteine is in line with a recent review on ambient air pollution and homocysteine, which reported a positive association between higher concentrations of PM 2.5 and/or PM10 and elevated homocysteine (Yang et al. 2020). However, our finding is contradictory to a recent longitudinal study (not included in the review above) on occupational exposure to welding fumes, where increased concentration levels of respirable dust were associated with decreased concentrations of homocysteine (Taj et al. 2021). However, in this study measurements of respirable dust and sampling of blood were not conducted on the same day, which may have affected the results. Welders have in earlier studies been associated with having increased risks of cardiovascular diseases, such as ischemic heart disease (Mocevic et al. 2015) and increased blood pressure (Li et al. 2015).

HDL concentrations are inversely, and LDL concentrations positively, associated with atherosclerosis and the resulting CVD. LDL have been established via both clinical and genetic studies to cause atherosclerosis (Ference et al. 2017). In tandem, HDL is necessary for preventing inflammatory process in atherosclerosis in the presence of high concentrations of LDL (Navab et al. 2011). In this study, we observed associations between higher exposure to respirable dust and dust of PM 0.1–10 with lower HDL concentrations. No increase in LDL concentrations was observed in relation to dust concentrations during work. After recovery during vacation, the HDL-concentrations did not differ markedly. However, LDL concentrations were reduced after vacation in all groups except those highly exposed to dust of PM 0.1–10 compared to LDL concentrations during work, indicating an association between exposure to dust and LDL. In addition, LDL concentrations were significantly higher (0.03 mmol/L) for each year exposed to dust. Altogether, these observations indicate that LDL-concentration is associated with both short- and long-term responses in relation to occupational exposure to dust. The overall increased LDL concentration along with long-term unchanged HDL concentration suggest an increased CVD risk. Dietary habits between the exposure groups were similar across exposure levels, which indicate that the observed associations with HDL and LDL are less likely to be confounded by dietary differences. Physical activity (Gordon et al. 2014) and alcohol consumption (Lamon-Fava 2002) could affect these results as physical activity (higher in all high exposed groups, Table 1) and moderate intake of alcohol (lower in all high exposed groups, Table 1) increase HDL levels. However, sensitivity analyses for all three types of dust adjusting for these two confounders showed minimal effects on the association between dust and HDL and LDL, respectively.

There are few studies on the association between particulate matter in occupational settings and metabolic biomarkers. In an Iranian study on occupational exposure to mineral dust and blood lipid parameters among workers in a ceramic tile factory, concentrations of triglycerides and HDL were found to be higher among the exposed as compared to the unexposed workers (Roshanaee et al. 2018). This finding is contradictory to our study’s results where higher exposure to respirable dust and dust of PM 0.1–10 were associated with lower HDL-levels. However, the Iranian study included smokers (and adjusted for smoking) and assumed similar dietary and lifestyle factors among both exposed and non-exposed workers based on self-reported statements on socio-economy; both factors could affect their results. In line with our findings is a Chinese study of ambient air pollution and blood lipids in adults, which found significant associations between exposure to PM1 and PM2.5 and higher concentrations of cholesterol, triglycerides and LDL, as well as lower concentrations of HDL (Yang et al. 2018). Also, another study of ambient air pollution in Taiwan and blood lipids in the general population observed associations between increased exposure to particulate matter < 10 µm and decreased levels of HDL (Chuang et al. 2010).

Biomarkers of systemic inflammation such as CRP and SAA are associated with risk of CVD (Danesh et al. 2000). Elevated concentrations of both CRP and SAA have been suggested to identify atherosclerosis and thus risk of CVD (Schillinger et al. 2005). However, within our study, there were no clear associations between exposure to respirable silica, respirable dust, or dust of PM 0.1–10 and CRP or SAA concentrations. This is not in line with a review that reported associations between different occupational and ambient particulate matter exposures and higher concentrations of CRP (Li et al. 2012). Furthermore, a systematic review and meta-analysis concluded that there is an association between ambient airborne particle exposure and increasing CRP-levels (Liu et al. 2019). One possible explanation for our results is that participants in all exposure groups did not report or even knew that they were having an ongoing infection at the time of blood sampling, which in turn may have blurred the associations with dust exposure.

Increases in particle exposure have previously been associated with blood pressure (Brook and Rajagopalan 2009; Giorginia et al. 2016; Regencia et al. 2021, 2020). Also, in our study, systolic blood pressure was significantly higher during work when the participants were exposed as compared to after vacation when they were non-exposed in all three high-exposed groups. Also, diastolic blood pressure was significantly elevated during work in the group high-exposed to respirable dust, though increased tendencies were seen also in workers high exposed to respirable silica and dust of PM 0.1–10. The reduction after vacation might also be due to participants having less stress during their vacation and/or exercised more (Byrne and Espnes 2008; Cornelissen and Smart 2013; Stewart et al. 2020).

All low-exposed groups had a significantly lower resting pulse rate during work than after vacation, but an opposite trend was seen in the high-exposed groups. It is possible that the measuring of blood pressure and pulse rate before vacation at the workplace vis-à-vis after vacation at the clinic could affect measurement due to white coat syndrome (Pioli et al. 2018). The white coat syndrome is a clinical condition which occurs when a person’s blood pressure levels are higher when measured by medical personnel compared to levels obtained by themselves. Earlier studies have suggested that anxiety increases levels of blood pressure and heart rate (Ogedegbe et al. 2008; Pioli et al. 2018). Also, the effect of dust exposure on the resting pulse rate could partly have diminished during vacation with decreased exposure. Since the effect of dust on resting pulse rate during work was strongest among highly exposed, the effect might only have remained after vacation in the high-exposed groups.

There is also a possibility of selection bias, that is, workers who decided to participate in the study did so because they suffered from health problems and potentially were seeking an association with their occupational dust exposure. However, the outcomes measured in this study (biomarkers and blood pressure) are often not known to the participants. We also asked about prior severe diseases and saw no differences between groups. Therefore, we consider these variables less likely to suffer from selection bias. Due to the recruitment process, it was only possible to calculate response rates among companies and not among workers.

Study strengths include performing state-of-the art, 6–8 h, individual sampling of several occupational exposures, as well as biological sampling on the same day as the exposure measurements. Furthermore, biological samples were measured longitudinally, during work and after vacation, thus allowing the study of recovery effects during vacation. Serum and plasma samples were handled in a homogeneous way and randomized before analysis, which minimizes bias due to batch effects or other technical errors. Also, we applied inclusion criteria to minimize some of the most common confounders that is excluding current daily smokers and restricting the sample to men only. Furthermore, we assessed concurrent occupational exposures such as noise, diesel fumes, or welding fumes, which did not significantly differ between workers exposed to low and high levels of dust. Thus, the occupational exposure that differed was the particle exposure under study.

Limitations of the present study relate to the relatively small sample size of 65 study participants which can affect statistical power. Quantitative measurements enabled continuous variables of both exposures and outcomes and thereby setting cut-offs which included enough participants in both the high- and low-exposed groups to maximize statistical power while retaining a sufficient contrast in exposure. Another limitation is that due to the inclusion criteria we have not studied those who have left their jobs possibly due to potential health aspects (healthy worker survivor effect) (Arrighi and Hertzpicciotto 1994). However, this could lead to lower associations between dust and the markers within (especially) the high-exposed groups, and thus an underestimation of the association. A further limitation involves exposure measurements being performed during one working day only. This means that the measures should be considered as crude indicators of average exposure levels for each participant. Most study participants had remained within the same worksite for several weeks, and it seems likely that the exposure levels were similar in the weeks prior to exposure measurements.

## Conclusion

In conclusion, our study indicates that exposure to dust may be associated with higher homocysteine concentrations, lower HDL concentrations and higher resting pulse rate. Homocysteine and LDL levels were also positively correlated to number of years of dust exposure, but no clear recovery effect after vacation was seen for homocysteine, indicating a long-term effect of exposure. Since effects were seen at mean exposure levels below the Swedish OEL for respirable silica (0.1 mg/m^3^) and respirable inorganic dust (2.5 mg/m^3^), a reduction of these occupational exposures may decrease the risk of cardiovascular diseases among exposed workers. However, more research is needed to confirm these findings in future studies.

## Supplementary Information

Below is the link to the electronic supplementary material.

Supplementary file1 (DOCX 131 KB)

## Data Availability

The datasets analyzed during the current study are not publicly available due to reasons of sensitivity, e.g. human data, but are available from the corresponding author on reasonable request.
