# Supplementary material for: Occupational exposure to particles and biomarkers of cardiovascular disease—during work and after vacation
Source: Int Arch Occup Environ Health. 2022 Jul 11;95(7):1537–48. doi: 10.1007/s00420-022-01900-5 (PMC9424160; doi:10.1007/s00420-022-01900-5)
Supplement: Supplementary file 1 — Supplementary file1 (DOCX 131 KB) [file 420_2022_1900_MOESM1_ESM.docx]

**International Archives of Occupational and Environmental Health**

**Supplementary Material
Occupational exposure to particles and biomarkers of cardiovascular disease – during work and after vacation**

Karin Grahn^1,2^, Karin Broberg^1,3^, Per Gustavsson^1,2^, Petter Ljungman^1,4^, Petra Lindfors^5^, Mattias Sjöström^1,2^, Pernilla Wiebert^1,2^, Jenny Selander^1^

^1)^ Institute of Environmental Medicine, Karolinska Institutet, Stockholm, Sweden. E-mail: [karin.grahn@ki.se](mailto:karin.grahn@ki.se)
^2)^ Centre for Occupational and Environmental Medicine, Region Stockholm, Stockholm, Sweden
^3)^ Division of Occupational and Environmental Medicine, Lund University, Lund, Sweden
^4)^ Department of Cardiology, Danderyd University Hospital, Danderyd, Sweden
^5)^ Department of Psychology, Stockholm University, Stockholm, Sweden

**Table S1** Health status of study participants dichotomized based on *a priori* categorized occupational groups carpenters or concrete and demolition workers. Number and proportion of participants with blood pressure, pulse and biomarkers deviating from reference-values.

**Table S2** Characteristics of study participants, all study participants and stratified for working in *a priori* categorized occupational groups carpenters or concrete and demolition workers ^a^. Percent (%) calculated from complete responses.

**Table S3** Effect estimates expressed as β-values with 95 % confidence intervals (CI) evaluated by linear regression analysis of all workers and adjusted for age, body mass index, alcohol consumption and physical activity. Based on measurements during work. For respirable silica, respirable dust, and dust of PM 0.1-10, respectively

**Table S4** Effect estimates expressed as β-values with 95 % confidence interval (CI) evaluated by linear regression, low group used as reference: blood pressure, pulse, and biomarkers in low- and high-exposed groups at measurements during work. The groups are dichotomized based on exposure measurements’ 50^th^ and 75^th^ percentile concentration levels, for respirable silica, respirable dust, and dust of PM 0.1-10, respectively

**Table S5** Sensitivity analyses with adjustments for age, BMI, and common viral infections or CVD-related drugs at measurements during work

**Table S6** Sensitivity analyses of the two sampling occasions/after vacation, dichotomized groups based on exposure measurements’ 75^th^ percentile concentration levels of respirable silica (0.026 mg/m^3^), respirable dust (0.462 mg/m^3^) and dust of PM 0.1-10 (0.984 mg/m^3^), respectively **a** including study participants not having any infection at any of the two sampling occasions
**b** including study participants not medicating with any CVD-related drug at any of the two sampling occasions
**c** including study participants not having worked prior to measurements after vacation

**Table S7** Effect estimates expressed as β-values with 95 % confidence interval (CI) of blood pressure, pulse and biomarkers in study participants associated with years of work in dust-exposed jobs, adjusted for BMI, and BMI and age (years), respectively. Linear regression with continuous variables.

**Table S1** Health status of study participants dichotomized based on *a priori* categorized occupational groups carpenters or concrete and demolition workers ^a^. Number and proportion of participants with blood pressure, pulse and biomarkers deviating from reference-values.

|  |  |  | **Carpenters**  ***n*=36** | |  | **Concrete and demolition workers**  ***n*=29** | |
| --- | --- | --- | --- | --- | --- | --- | --- |
|  | ***Ref. value*** |  | *n* | % |  | *n* | % |
| Systolic BP (mm Hg) | < 140 |  | 6 | 17 |  | 4 | 14 |
| Diastolic BP (mm Hg) | < 90 |  | 9 | 25 |  | 7 | 24 |
| C-reactive protein (mg/L) | < 3 |  | 3 | 8 |  | 3 | 10 |
| Homocystein (µmol/L) | < 15 |  | 10 | 28 |  | 8 | 29 |
| Cholesterol (mmol/L) |  |  | 2 | 6 |  | 3 | 10 |
| Cholesterol (mmol/L) *<= 30 years* | 2.9-6.1 |  | 0 | 0 |  | 0 | 0 |
| Cholesterol (mmol/L) *31-49 years* | 3.3-6.9 |  | 0 | 0 |  | 1 | 3 |
| Cholesterol (mmol/L) *>= 50 years* | 3.9-7.8 |  | 2 | 6 |  | 1 | 3 |
| High density lipoprotein (mmol/L) *men* | 0.8 - 2.1 |  | 3 | 8 |  | 1 | 3 |
| Low density lipoprotein (mmol/L) |  |  | 2 | 6 |  | 3 | 10 |
| Low density lipoprotein (mmol/L) *<= 30 years* | 1.2-4.3 |  | 0 | 0 |  | 0 | 0 |
| Low density lipoprotein (mmol/L) *31-49 years* | 1.4-4.7 |  | 0 | 0 |  | 0 | 0 |
| Low density lipoprotein (mmol/L) *>= 50 years* | 2.0-5.3 |  | 2 | 6 |  | 3 | 10 |
| Triglycerides (mmol/L) *>= 18 years* | 0.4 - 2.6 |  | 2 | 6 |  | 4 | 14 |
| Pulse, resting (bpm) |  |  |  |  |  |  |  |
| Serum Amyloid A (mg/L) | < 6.4 |  | 4 | 11 |  | 5 | 17 |
| Fibrinogen (g/L) | 2.0 - 4.0 |  | 3 | 8 |  | 2 | 7 |

^a^ Carpenters: 35 construction carpenters, and 1 plumber; Concrete and demolition workers: 10 concrete/stone drillers, 10 demolition workers, 6 concrete workers, 2 plasterers, and 1 bricklayer

**Table S2** Characteristics of study participants, all study participants and stratified for working in *a priori* categorized occupational groups carpenters or concrete and demolition workers ^a^. Percent (%) calculated from complete responses.

|  |  | All  *n*=65 |  |  |  |  | Carpenters  *n*=36 |  |  |  |  | Concrete and demolition workers  *n*=29 |  |  |  |
| --- | --- | --- | --- | --- | --- | --- | --- | --- | --- | --- | --- | --- | --- | --- | --- |
| **Individual risk factors** | *Mean* | *Min-Max* | *n* | *%* |  | *Mean* | *Min-Max* | *n* | *%* |  | *Mean* | *Min-Max* | *n* | *%* |  |
| Age (years) | 39.7 | 20.0-65.0 |  |  |  | 41.1 | 20.0-65.0 |  |  |  | 38.0 | 21.0-61.0 |  |  |  |
| BMI (kg/m^2^) ^b^ | 27.9 | 19.3-45.9 |  |  |  | 27.7 | 19.0-40.0 |  |  |  | 28.0 | 20.0-46.0 |  |  |  |
| Ever smoker (yes) ^c^ |  |  | 26 | 41.3 |  |  |  | 14 | 40 |  |  |  | 12 | 43 |  |
| Ever smoking (years) | 11.4 | 1.0-26.0 |  |  |  | 9.7 | 3.0-21.0 |  |  |  | 13.5 | 1.0-26.0 |  |  |  |
| Alcohol consumption (>= 4 times/week) |  |  | 12 | 18.8 |  |  |  | 8 | 23 |  |  |  | 4 | 14 |  |
| Vegetable consumption (>= 5 times/week) |  |  | 44 | 68.8 |  |  |  | 25 | 71 |  |  |  | 19 | 66 |  |
| Physical activity (high) ^d^ |  |  | 30 | 46.9 |  |  |  | 20 | 56 |  |  |  | 10 | 36 |  |
|  |  |  |  |  |  |  |  |  |  |  |  |  |  |  |  |
| **Occupational risk factors** |  |  |  |  |  |  |  |  |  |  |  |  |  |  |  |
| Years in dusty profession | 14.1 | 0-43 |  |  |  | 16.3 | 0-40 |  |  |  | 11.2 | 0-43 |  |  |  |
| Respiratory mask use (yes) ^e^ |  |  | 11 | 16.9 |  |  |  | 1 | 3 |  |  |  | 10 | 34 |  |
| Noise (yes) |  |  | 64 | 100.0 |  |  |  | 35 | 100 |  |  |  | 29 | 100 |  |
| Hearing protector use (yes) ^f^ |  |  | 61 | 93.8 |  |  |  | 35 | 97 |  |  |  | 26 | 90 |  |
| Vibration whole-body (yes) |  |  | 31 | 47.7 |  |  |  | 13 | 36 |  |  |  | 18 | 62 |  |
| Vibration hand/arm (yes) |  |  | 61 | 96.8 |  |  |  | 33 | 94 |  |  |  | 28 | 100 |  |
| Working in cold temperature (yes) |  |  | 58 | 89.2 |  |  |  | 31 | 86 |  |  |  | 27 | 93 |  |
| Diesel fumes (yes) |  |  | 29 | 89.2 |  |  |  | 18 | 50 |  |  |  | 11 | 38 |  |
| Chemical vapours/gases (yes) |  |  | 23 | 36.5 |  |  |  | 15 | 42 |  |  |  | 8 | 30 |  |
| Welding fumes (yes) |  |  | 21 | 32.8 |  |  |  | 15 | 42 |  |  |  | 6 | 21 |  |
| Dust other than silica (yes) |  |  | 56 | 90.3 |  |  |  | 32 | 94 |  |  |  | 24 | 86 |  |
| Physically demanding work (yes) |  |  | 62 | 96.9 |  |  |  | 35 | 97 |  |  |  | 27 | 93 |  |
| Psychologically demanding work (yes) |  |  | 38 | 63.3 |  |  |  | 21 | 58 |  |  |  | 17 | 59 |  |
| Exposure RCS ^g^ from hobby (yes) |  |  | 12 | 18.8 |  |  |  | 8 | 22 |  |  |  | 4 | 14 |  |
|  |  |  |  |  |  |  |  |  |  |  |  |  |  |  |  |
| **Diseases** |  |  |  |  |  |  |  |  |  |  |  |  |  |  |  |
| Any CVD-related disease (yes) |  |  | 17 | 27.9 |  |  |  | 10 | 29.4 |  |  |  | 7 | 25.9 |  |
| Myocardial infarction (yes) |  |  | 2 | 3.2 |  |  |  | 1 | 2.8 |  |  |  | 1 | 3.4 |  |
| Angina pectoris (yes) |  |  | 4 | 6.2 |  |  |  | 1 | 2.8 |  |  |  | 3 | 10.3 |  |
| Hypertension (yes) |  |  | 14 | 21.9 |  |  |  | 10 | 28.6 |  |  |  | 4 | 13.8 |  |
| Stroke (yes) |  |  | 0 | N.A. |  |  |  | 0 | 0.0 |  |  |  | 0 | 0.0 |  |
| Thrombosis in arm/leg (yes) |  |  | 1 | 1.6 |  |  |  | 1 | 2.8 |  |  |  | 0 | 0.0 |  |
| Other heart disease (yes) |  |  | 2 | 3.1 |  |  |  | 1 | 2.9 |  |  |  | 1 | 3.4 |  |
| Kidney/urinary disease (yes) |  |  | 2 | 3.1 |  |  |  | 1 | 2.9 |  |  |  | 1 | 3.4 |  |
| Diabetes (yes) |  |  | 3 | 4.7 |  |  |  | 2 | 5.7 |  |  |  | 1 | 3.4 |  |
| Family history of myocardial infarction (yes) |  |  | 8 | 12.3 |  |  |  | 7 | 19.4 |  |  |  | 1 | 3.4 |  |
| Family history of stroke (yes) |  |  | 2 | 3.1 |  |  |  | 2 | 5.7 |  |  |  | 0 | 0.0 |  |
| Family history of hypertension (yes) |  |  | 18 | 27.7 |  |  |  | 10 | 27.8 |  |  |  | 8 | 27.6 |  |
|  |  |  |  |  |  |  |  |  |  |  |  |  |  |  |  |
| **Medicine** |  |  |  |  |  |  |  |  |  |  |  |  |  |  |  |
| Use prescribed medicine (yes) |  |  | 12 | 18.5 |  |  |  | 9 | 25.0 |  |  |  | 3 | 10.3 |  |
| Use non-prescribed medicine (yes) |  |  | 15 | 23.1 |  |  |  | 7 | 19.4 |  |  |  | 8 | 27.6 |  |

^a^ Carpenters: 35 construction carpenters, and 1 plumber; Concrete and demolition workers: 10 concrete/stone drillers, 10 demolition workers, 6 concrete workers, 2 plasterers, and 1 bricklayer ^b^ Body mass index, calculated from height and weight measurements
^c^ Including 4 participants who are party-smokers (1 in the occupational group carpenters, 3 in the occupational group concrete and demolition workers)
^d^ Once a week or more of minimum 30 min regular physical activity
^e^ Observed usage during day of measurements. Type of respirator mask: filter or air-supplied
^f^ Observed usage during day of measurements. Type of hearing protection: ear muffs or ear plugs
^g^ Respirable crystalline silica

**Table S3** Effect estimates expressed as β-values with 95 % confidence intervals (CI) evaluated by linear regression analysis of all workers and adjusted for age, body mass index, alcohol consumption and physical activity. Based on measurements during work. For respirable silica, respirable dust, and dust of PM 0.1-10, respectively. The β represents the change in each variable with an increase in exposure of 1 mg/m^3^.

|  |  | **Respirable silica**  **continuous variables** | |  | **Respirable dust**  **continuous variables** | |  | **Dust of PM 0.1-10**  **continuous variables** | |
| --- | --- | --- | --- | --- | --- | --- | --- | --- | --- |
|  |  | Adj  β ^a^ | 95 % CI |  | Adj  β ^a^ | 95 % CI |  | Adj  β ^a^ | 95 % CI |
| Systolic BP (mm Hg) |  | -15.85 | -108.55, 76.83 |  | 0.74 | -7.87, 9.36 |  | 1.40 | -2.51, 5.31 |
| Diastolic BP (mm Hg) |  | -12.80 | -85.28, 59.68 |  | 1.13 | -5.60, 7.86 |  | 1.00 | -1.69, 3.69 |
| CRP (mg/L) |  | -0.73 | -20.48, 19.02 |  | 0.62 | -1.20, 2.45 |  | 0.18 | -0.63, 0.99 |
| Homocystein (umol/L) ^b^ |  | 21.48 | -0.85, 43.81 |  | 1.80 | -0.29, 3.89 |  | 1.09 | 0.10, 2.08 |
| Cholesterol (mmol/L) |  | -1.14 | -7.54, 5.26 |  | -0.20 | -0.80, 0.39 |  | -0.10 | -0.37, 0.17 |
| HDL (mmol/L) |  | -0.95 | -3.08, 1.19 |  | -0.20 | -0.39, -0.00 |  | -0.08 | -0.17, 0.01 |
| LDL (mmol/L) |  | -0.22 | -5.75, 5.30 |  | -0.13 | -0.64, 0.38 |  | -0.03 | -0.27, 0.20 |
| Triglycerides (mmol/L) |  | -1.57 | -10.64, 7.50 |  | -0.02 | -0.86, 0.83 |  | -0.02 | -0.46, 0.41 |
| Pulse, resting (bpm) |  | 17.09 | -53.12, 87.31 |  | 7.69 | 1.49, 13.90 |  | 3.45 | 0.68, 6.22 |
| s_saa |  | -5.09 | -26.26, 16.09 |  | -0.49 | -2.45, 1.48 |  | 0.01 | -0.85, 0.88 |
| fibrogen |  | 1.18 | -2.35, 4.71 |  | 0.11 | -0.22, 0.43 |  | 0.06 | -0.10, 0.22 |

^a^ Linear regression, adjusted for age, body mass index (BMI), alcohol consumption (high: > 4 times per week/low: ≤ 4 times per week), and physical activity (high: ≥ once a week or more of minimum 30 min regular physical activity)/low: < once a week or more of minimum 30 min regular physical activity)
^b^ 1 missing

**Table S4** Effect estimates expressed as β-values with 95 % confidence interval (CI) evaluated by linear regression, low group used as reference: blood pressure, pulse, and biomarkers in low- and high-exposed groups at measurements during work. The groups are dichotomized based on exposure measurements’ 50^th^ and 75^th^ percentile concentration levels, for respirable silica, respirable dust, and dust of PM 0.1-10, respectively. The β represents the difference of each variable between the two groups, using the low group as a reference.

|  |  | **50^th^ percentile** |  |  |  |  | **75^th^ percentile** |  |  |
| --- | --- | --- | --- | --- | --- | --- | --- | --- | --- |
|  | Crude  β | 95 % CI | Adj  β ^a^ | 95 % CI |  | Crude  β | 95 % CI | Adj  β ^a^ | 95 % CI |
| **Respirable silica ^b^** |  |  |  |  |  |  |  |  |  |
| Systolic blood pressure (mm Hg) | -4.82 | -11.81, 2.17 | -1.67 | -8.59, 5.24 |  | -5.81 | -13.91, 2.30 | -2.54 | -10.59, 5.50 |
| Diastolic blood pressure (mm Hg) | -5.25 | -11.11, 0.61 | -1.66 | -7.24, 3.92 |  | -6.09 | -12.87, 0.73 | -1.98 | -8.49, 4.52 |
| C-reactive protein (mg/L) | -0.18 | -1.64, 1.27 | 0.38 | - 1.06, 1.83 |  | -0.99 | -2.66, 0.69 | -0.52 | -2.20, 1.16 |
| Homocystein (µmol/L) ^c^ | 0.84 | -0.85, 2.53 | 0.9 | -0.90, 2.70 |  | 2.04 | 0.14, 3.94 | 2.29 | 0.27, 4.32 |
| Cholesterol (mmol/L) | -0.40 | -0.88, 0.08 | -0.25 | -0.72, 0.22 |  | -0.41 | -0.97, 0.15 | -0.18 | -0.72, 0.37 |
| High density lipoprotein (mmol/L) | -0.01 | -0.18, 0.15 | -0.05 | -0.22, 0.11 |  | -0.06 | -0.25, 0.13 | -0.10 | -0.29, 0.09 |
| Low density lipoprotein (mmol/L) | -0.19 | - 0.60, 0.21 | -0.13 | - 0.54, 0.27 |  | -0.26 | -0.73, 0.22 | -0.15 | -0.62, 0.33 |
| Triglycerides (mmol/L) | -0.42 | -1.06, 0.22 | -0.21 | -0.87, 0.45 |  | -0.31 | -1.06, 0.44 | -0.06 | -0.83, 0.71 |
| Pulse, resting (bpm) | -2.74 | -7.99, 2.51 | -0.69 | -5.98, 4.60 |  | 0.98 | -5.17, 7.12 | 3.63 | -2.48, 9.73 |
| Serum Amyloid A (mg/L) | -0.78 | -2.38, 0.81 | -0.05 | -1.62, 1.53 |  | -1.59 | -3.41, 0.24 | -0.90 | -2.72, 0.93 |
| Fibrinogen (g/L) | -0.07 | -0.35, 0.21 | 0.09 | -0.17, 0.36 |  | -0.11 | -0.44, 0.21 | 0.06 | -0.25, 0.37 |
|  |  |  |  |  |  |  |  |  |  |
| **Respirable dust ^d^** |  |  |  |  |  |  |  |  |  |
| Systolic blood pressure (mm Hg) | -0.11 | -7.20, 6.99 | 1.76 | -4.90, 8.42 |  | -4.64 | -12.79, 3.50 | -1.18 | -9.10, 6.73 |
| Diastolic blood pressure (mm Hg) | 0.51 | - 5.50, 6.52 | 2.75 | -2.60, 8.10 |  | -4.77 | -11.64, 2.10 | -1.03 | -7.42, 5.37 |
| C-reactive protein (mg/L) | 0.75 | -0.70, 2.20 | 1.06 | -0.31, 2.43 |  | 0.52 | -1.16, 2.21 | 1.21 | -0.41, 2.84 |
| Homocystein (µmol/L) ^e^ | 1.48 | -0.19, 3.14 | 1.53 | -0.19, 3.25 |  | 3.38 | 1.61, 5.14 | 3.59 | 1.73, 5.44 |
| Cholesterol (mmol/L) | 0.02 | -0.47, 0.51 | 0.14 | -0.31, 0.60 |  | -0.20 | -0.77, 0.37 | -0.07 | -0.61, 0.47 |
| High density lipoprotein (mmol/L) | -0.09 | -0.25, 0.08 | -0.11 | -0.26, 0.05 |  | -0.12 | -0.31, 0.07 | -0.17 | -0.36, 0.01 |
| Low density lipoprotein (mmol/L) | -0.12 | - 0.53, 0.29 | -0.07 | -0.46, 0.32 |  | -0.01 | -0.49, 0.46 | 0.03 | -0.43, 0.49 |
| Triglycerides (mmol/L) | 0.24 | -0.40, 0.89 | 0.39 | -0.24, 1.02 |  | -0.21 | -0.96, 0.54 | 0.04 | -0.72, 0.80 |
| Pulse, resting (bpm) | 2.25 | -3.02, 7.52 | 3.52 | -1.50, 8.55 |  | 3.49 | -2.59, 9.58 | 6.27 | 0.43, 12.11 |
| Serum Amyloid A (mg/L) | 0.12 | -1.49, 1.73 | 0.54 | -0.98, 2.05 |  | -0.21 | -2.08, 1.65 | 0.63 | -1.17, 2.42 |
| Fibrinogen (g/L) | 0.08 | -0.20, 0.35 | 0.17 | - 0.09, 0.42 |  | 0.03 | -0.29, 0.36 | 0.21 | -0.08, 0.51 |
|  |  |  |  |  |  |  |  |  |  |
| **Dust of PM 0.1-10 ^f^** |  |  |  |  |  |  |  |  |  |
| Systolic blood pressure (mm Hg) | 5.68 | -1.50, 12.85 | 5.86 | -1.05, 12.76 |  | -1.48 | -9.91, 6.95 | 0.68 | -7.71, 9.06 |
| Diastolic blood pressure (mm Hg) | 4.42 | -1.34, 10.19 | 4.88 | -0.02, 9.78 |  | -1.17 | -7.93, 5.60 | 1.02 | -4.98, 7.03 |
| C-reactive protein (mg/L) | 0.75 | -0.70, 2.19 | 0.77 | -0.69, 2.22 |  | 1.45 | -0.18, 3.07 | 1.80 | 0.15, 3.45 |
| Homocystein (µmol/L) ^g^ | 2.12 | 0.28, 3.96 | 2.15 | 0.27, 4.03 |  | 3.53 | 1.50, 5.56 | 3.78 | 1.61, 5.94 |
| Cholesterol (mmol/L) | -0.13 | -0.69, 0.44 | -0.07 | -0.56, 0.43 |  | -0.05 | -0.70, 0.61 | 0.08 | -0.51, 0.66 |
| High density lipoprotein (mmol/L) | -0.05 | -0.25, 0.15 | -0.04 | -0.22, 0.14 |  | -0.12 | -0.34, 0.11 | -0.20 | -0.41, 0.01 |
| Low density lipoprotein (mmol/L) | -0.25 | -0.70, 0.20 | -0.21 | -0.63, 0.21 |  | -0.27 | -0.79, 0.25 | -0.25 | -0.74, 0.24 |
| Triglycerides (mmol/L) | 0.17 | -0.65, 0.99 | 0.17 | -0.61, 0.94 |  | 0.34 | -0.60, 1.28 | 0.69 | -0.20, 1.59 |
| Pulse, resting (bpm) | 6.32 | 1.11, 11.52 | 6.45 | 1.29, 11.60 |  | 5.62 | -0.49, 11.73 | 7.08 | 0.95, 13.21 |
| Serum Amyloid A (mg/L) | 0.59 | -1.00, 2.18 | 0.57 | -0.98, 2.12 |  | 0.65 | -1.18, 2.47 | 1.19 | -0.62, 2.99 |
| Fibrinogen (g/L) | 0.05 | -0.26, 0.37 | 0.05 | -0.25, 0.35 |  | 0.19 | -0.16, 0.55 | 0.32 | -0.02, 0.66 |

^a^ Linear regression, adjusted for age and body mass index (BMI)
^b^ Respirable silica; 50^th^ percentile: 0.015 mg/m^3^, 75^th^ percentile: 0.026 mg/m^3^
^c^ 1 missing in the low-exposed group both for 50^th^ and 75^th^ percentile
^d^ Respirable dust; 50^th^ percentile: 0.249 mg/m^3^, 75^th^ percentile: 0.462 mg/m^3^
^e^ 1 missing in the high-exposed group for 50^th^ percentile, and 1 missing in the low-exposed group for 75^th^ percentile ^f^ Dust of PM 0.1-10; 50^th^ percentile: 0.473 mg/m^3^, 75^th^ percentile: 0.984 mg/m^3^
^g^ 1 missing in the high-exposed group both for the 50^th^ and 75^th^ percentile

**Table S5** Sensitivity analyses with adjustments for age, BMI, and common viral infections or CVD-related drugs at measurements during work. Effect estimates expressed as β-values with 95 % confidence interval (CI) evaluated by linear regression, continuous variables. The β represents the difference of each variable with 1 mg/m^3^ increase of exposure.

|  | **Infection** | |  | **CVD-drug** | |
| --- | --- | --- | --- | --- | --- |
|  | Adj  β ^a^ | 95 % CI |  | Adj  β ^b^ | 95 % CI |
| **Respirable silica** |  |  |  |  |  |
| Systolic blood pressure (mm Hg) | -48.32 | -134.92, 38.29 |  | -12.81 | -101.80, 76.18 |
| Diastolic blood pressure (mm Hg) | -43.28 | -112.92, 26.37 |  | -13.57 | -85.62, 58.47 |
| C-reactive protein (mg/L) | -0.86 | -20.27, 18.55 |  | 0.66 | -18.04, 19.35 |
| Homocystein (µmol/L) ^c^ | 24.58 | 1.23, 47.92 |  | 25.28 | 2.83, 47.73 |
| Cholesterol (mmol/L) | -0.18 | -6.28, 5.92 |  | -2.14 | -7.92, 3.64 |
| High density lipoprotein (mmol/L) | -1.19 | -3.39, 1.01 |  | -1.11 | -3.22, 1.01 |
| Low density lipoprotein (mmol/L) | 0.32 | -5.02, 5.66 |  | -0.97 | -5.96, 4.03 |
| Triglycerides (mmol/L) | -0.40 | -9.25, 8.45 |  | -1.59 | -10.14, 6.95 |
| Pulse, resting (bpm) | 6.44 | -61.20, 74.08 |  | 28.88 | -39.18, 96.95 |
| Serum Amyloid A (mg/L) | -0.04 | -21.22, 21.13 |  | -1.54 | -21.86, 18.78 |
| Fibrinogen (g/L) | 1.03 | -2.46, 4.51 |  | 1.61 | -1.78, 5.00 |
|  |  |  |  |  |  |
| **Respirable dust** |  |  |  |  |  |
| Systolic blood pressure (mm Hg) | -1.25 | -9.21, 6.71 |  | 0.72 | -7.57, 9.01 |
| Diastolic blood pressure (mm Hg) | -0.77 | -7.19, 5.65 |  | 0.88 | -5.83, 7.59 |
| C-reactive protein (mg/L) | 0.60 | -1.16, 2.36 |  | 0.66 | -1.07, 2.40 |
| Homocystein (µmol/L) ^c^ | 2.16 | 0.02, 4.30 |  | 2.25 | 0.15, 4.35 |
| Cholesterol (mmol/L) | -0.16 | -0.71, 0.40 |  | -0.30 | -0.84, 0.23 |
| High density lipoprotein (mmol/L) | -0.23 | -0.42, -0.04 |  | -0.22 | -0.41, -0.03 |
| Low density lipoprotein (mmol/L) | -0.11 | -0.60, 0.37 |  | -0.21 | -0.67, 0.25 |
| Triglycerides (mmol/L) | 0.08 | -0.72, 0.89 |  | -0.01 | -0.80, 0.79 |
| Pulse, resting (bpm) | 6.88 | 0.98, 12.78 |  | 8.13 | 2.11, 14.15 |
| Serum Amyloid A (mg/L) | -0.01 | -1.94, 1.92 |  | -0.13 | -2.02, 1.76 |
| Fibrinogen (g/L) | 0.11 | -0.21, 0.43 |  | 0.15 | -0.17, 0.46 |
|  |  |  |  |  |  |
| **Dust of PM 0.1-10** |  |  |  |  |  |
| Systolic blood pressure (mm Hg) | 1.08 | -2.64, 4.80 |  | 1.72 | -2.08, 5.52 |
| Diastolic blood pressure (mm Hg) | 0.79 | -1.87, 3.45 |  | 1.36 | -1.36, 4.08 |
| C-reactive protein (mg/L) | 0.28 | -0.52, 1.08 |  | 0.21 | -0.58, 1.00 |
| Homocystein (µmol/L) ^c^ | 1.14 | 0.13, 2.15 |  | 1.21 | 0.21, 2.21 |
| Cholesterol (mmol/L) | -0.05 | -0.31, 0.20 |  | -0.15 | -0.40, 0.10 |
| High density lipoprotein (mmol/L) | -0.10 | -0.19, 0.00 |  | -0.10 | -0.19, -0.00 |
| Low density lipoprotein (mmol/L) | -0.01 | -0.23, 0.22 |  | -0.07 | -0.29, 0.15 |
| Triglycerides (mmol/L) | 0.03 | -0.39, 0.45 |  | -0.02 | -0.44, 0.40 |
| Pulse, resting (bpm) | 3.22 | 0.56, 5.88 |  | 3.80 | 1.06, 6.54 |
| Serum Amyloid A (mg/L) | 0.12 | -0.73, 0.97 |  | 0.04 | -0.79, 0.87 |
| Fibrinogen (g/L) | 0.05 | -0.11, 0.21 |  | 0.07 | -0.09, 0.23 |

^a^ Linear regression, adjusted for age, body mass index (BMI), and infection
^b^ Linear regression, adjusted for age, body mass index (BMI), and CVD-related drug
^c^ 1 missing

**Table S6a** Sensitivity analyses including study participants not having any infection at any of the two sampling occasions (measurements during work and after vacation): changes in blood pressure, pulse, and biomarkers in low- and high-exposed groups. The groups are dichotomized based on exposure measurements’ 75^th^ percentile concentration levels of respirable silica (0.026 mg/m^3^), respirable dust (0.462 mg/m^3^) and dust of PM 0.1-10 (0.984 mg/m^3^), respectively. A positive value indicates higher point estimates during work than after vacation.

| **No infection** | ***n*** | **Mean  of change** | **IQR ^a^** | **Min** | **Max** | **p-value ^b^** |  | ***n*** | **Mean  of change** | **IQR ^a^** | **Min** | **Max** | **p-value ^b^** |
| --- | --- | --- | --- | --- | --- | --- | --- | --- | --- | --- | --- | --- | --- |
| **75^th^ percentile (0.026 mg/m^3^) of respirable silica** |  |  | Low, *n* = 44 |  |  |  |  |  |  | High, *n* = 12 |  |  |  |
| Systolic blood pressure (mm Hg) | 42 | -0.28 | -5.33 5.83 | -20.50 | 15.83 | 0.83 |  | 12 | 3.89 | 2.17 7.17 | -7.67 | 10.00 | 0.02 |
| Diastolic blood pressure (mm Hg) | 42 | -0.63 | -5.00 5.33 | 16.33 | 12.67 | 0.58 |  | 12 | 3.64 | -2.67 10.00 | -8.67 | 15.33 | 0.11 |
| C-reactive protein (mg/L) | 42 | -0.21 | -0.62 0.38 | -6.30 | 7.50 | 0.49 |  | 12 | -0.92 | -2.00 -0.06 | -3.34 | 1.19 | 0.05 |
| Homocystein (µmol/L) | 41 | -0.22 | -1.00 1.00 | -10.00 | 8.00 | 0.67 |  | 12 | -0.50 | -2.00 2.00 | -5.00 | 4.00 | 0.56 |
| Cholesterol (mmol/L) | 42 | -0.07 | -0.40 0.30 | -1.60 | 1.60 | 0.49 |  | 12 | -0.19 | -0.60 0.15 | -1.00 | 0.50 | 0.20 |
| High density lipoprotein (mmol/L) | 41 | -0.03 | -0.10 0.10 | -0.50 | 0.40 | 0.36 |  | 12 | -0.02 | -0.15 0.10 | -0.20 | 0.21 | 0.59 |
| Low density lipoprotein (mmol/L) | 42 | 1.32 | 0.90 2.00 | -8.90 | 3.60 | <0.01 |  | 12 | 1.37 | 0.85 1.68 | 0.30 | 2.70 | <0.01 |
| Triglycerides (mmol/L) | 42 | -0.15 | -0.50 0.40 | -4.90 | 0.90 | 0.34 |  | 12 | -0.13 | - 0.40 0.00 | -1.00 | 0.90 | 0.38 |
| Pulse, resting (bpm) | 42 | -4.21 | -8.67 1.00 | -23.33 | 19.00 | <0.01 |  | 12 | 4.69 | -2.83 11.83 | -11.67 | 21.00 | 0.14 |
| Serum Amyloid A (mg/L) | 42 | -0.49 | -0.75 0.68 | -13.78 | 10.71 | 0.44 |  | 12 | -2.11 | -1.52 0.36 | -12.15 | 1.03 | 0.14 |
| Fibrinogen (g/L) | 42 | 0.04 | -0.30 0.20 | -1.50 | 1.00 | 0.62 |  | 11 | -0.07 | -0.40 0.40 | -1.10 | 1.00 | 0.69 |
|  |  |  |  |  |  |  |  |  |  |  |  |  |  |
| **75^th^ percentile (0.462** **mg/m^3^) of respirable dust** |  |  | Low, *n* = 42 |  |  |  |  |  |  | High, *n* = 14 |  |  |  |
| Systolic blood pressure (mm Hg) | 41 | -0.78 | -5.33 4.50 | -20.50 | 15.83 | 0.56 |  | 13 | 5.14 | 4.00 7.33 | -7.67 | 12.17 | <0.01 |
| Diastolic blood pressure (mm Hg) | 41 | -0.98 | -5.00 5.00 | -16.33 | 12.67 | 0.40 |  | 13 | 4.41 | 1.67 9.67 | -8.67 | 15.33 | 0.04 |
| C-reactive protein (mg/L) | 41 | -0.12 | -0.62 0.38 | -6.30 | 7.50 | 0.68 |  | 13 | -1.14 | -2.70 0.00 | -4.72 | 1.19 | 0.04 |
| Homocystein (µmol/L) | 40 | -0.48 | -1.00 1.00 | -10.00 | 8.00 | 0.34 |  | 13 | 0.31 | -1.00 3.00 | -5.00 | 4.00 | 0.73 |
| Cholesterol (mmol/L) | 41 | -0.12 | -0.50 0.20 | -1.60 | 1.10 | 0.18 |  | 13 | 0.00 | -0.40 0.20 | -1.00 | 1.60 | 1.00 |
| High density lipoprotein (mmol/L) | 40 | -0.03 | -0.15 0.10 | -0.50 | 0.40 | 0.30 |  | 13 | -0.01 | -0.10 0.10 | -0.20 | 0.21 | 0.84 |
| Low density lipoprotein (mmol/L) | 41 | 1.27 | 0.90 2.00 | -8.90 | 3.20 | <0.01 |  | 13 | 1.53 | 0.90 1.70 | 0.30 | 3.60 | <0.01 |
| Triglycerides (mmol/L) | 41 | -0.18 | -0.50 0.30 | -4.90 | 1.40 | 0.25 |  | 13 | -0.03 | -0.40 0.00 | -1.00 | 1.10 | 0.85 |
| Pulse, resting (bpm) | 41 | -4.60 | -8.67 0.33 | -23.33 | 19.00 | <0.01 |  | 13 | 5.23 | -2.00 10.67 | -11.67 | 21.00 | 0.07 |
| Serum Amyloid A (mg/L) | 41 | -0.20 | -0.73 0.43 | -11.74 | 10.71 | 0.72 |  | 13 | -2.90 | -1.59 0.41 | -13.78 | 1.17 | 0.08 |
| Fibrinogen (g/L) | 41 | 0.06 | -0.30 0.20 | -0.70 | 1.00 | 0.35 |  | 12 | -0.15 | -0.40 0.25 | -1.50 | 1.00 | 0.47 |
|  |  |  |  |  |  |  |  |  |  |  |  |  |  |
| **75^th^ percentile (0.984 mg/m^3^) of dust of PM 0.1-10** |  |  | Low, *n* = 33 |  |  |  |  |  |  | High, *n* =10 |  |  |  |
| Systolic blood pressure (mm Hg) | 32 | -0.42 | -6.75 7.25 | -20.50 | 15.83 | 0.80 |  | 9 | 4.11 | 3.67 5.50 | -0.50 | 7.33 | <0.01 |
| Diastolic blood pressure (mm Hg) | 32 | -0.92 | -5.17 5.50 | -16.33 | 12.67 | 0.50 |  | 9 | 3.19 | 2.67 8.67 | -8.67 | 10.33 | 0.18 |
| C-reactive protein (mg/L) | 32 | -0.48 | -0.83 0.23 | -6.30 | 1.60 | 0.06 |  | 9 | -1.67 | -3.10 -0.24 | -4.72 | 1.19 | 0.03 |
| Homocystein (µmol/L) ^c^ | 32 | -0.63 | -1.50 1.00 | -10.00 | 8.00 | 0.32 |  | 8 | 0.00 | -3.00 3.00 | -5.00 | 4.00 | 1.00 |
| Cholesterol (mmol/L) | 32 | -0.12 | -0.55 0.25 | -0.90 | 0.90 | 0.21 |  | 9 | 0.19 | -0.40 0.50 | -1.00 | 1.60 | 0.52 |
| High density lipoprotein (mmol/L) | 32 | -0.03 | -0.10 0.10 | -0.50 | 0.40 | 0.50 |  | 8 | 0.01 | -0.15 0.10 | -0.20 | 0.21 | 0.81 |
| Low density lipoprotein (mmol/L) | 32 | 1.50 | 1.05 1.91 | -0.10 | 3.20 | <0.01 |  | 9 | 0.50 | 0.80 1.70 | -8.90 | 3.60 | 0.69 |
| Triglycerides (mmol/L) | 32 | -0.07 | -0.60 0.35 | -1.80 | 1.40 | 0.59 |  | 9 | -0.52 | -0.40 0.00 | -4.90 | 1.10 | 0.39 |
| Pulse, resting (bpm) | 32 | -5.78 | -10.33 0.67 | -23.33 | 10.67 | <0.01 |  | 9 | 4.70 | -3.00 13.00 | -11.67 | 21.00 | 0.27 |
| Serum Amyloid A (mg/L) | 32 | -0.91 | -0.84 0.18 | -11.74 | 6.70 | 0.11 |  | 9 | -4.40 | -11.23 -0.28 | -13.78 | 1.03 | 0.06 |
| Fibrinogen (g/L) | 31 | -0.03 | -0.40 0.20 | -0.70 | 0.80 | 0.70 |  | 9 | -0.09 | -0.30 0.40 | -1.50 | 1.00 | 0.75 |

**^a^** Inter-quartile range
**^b^** Paired t-test

**Table S6b** Sensitivity analyses including study participants not medicating with any CVD-related drug at any of the two sampling occasions (measurements during work and after vacation): changes in blood pressure, pulse, and biomarkers in low- and high-exposed groups. The groups are dichotomized based on exposure measurements’ 75^th^ percentile concentration levels of respirable silica (0.026 mg/m^3^), respirable dust (0.462 mg/m^3^) and dust of PM 0.1-10 (0.984 mg/m^3^), respectively. A positive value indicates higher point estimates during work than after vacation.

| **No CVD-related drugs** | ***n*** | **Mean of change** | **IQR ^a^** | **Min** | **Max** | **p-value ^b^** |  | ***n*** | **Mean of change** | **IQR ^a^** | **Min** | **Max** | **p-value ^b^** |
| --- | --- | --- | --- | --- | --- | --- | --- | --- | --- | --- | --- | --- | --- |
| **75^th^ percentile (0.026 mg/m^3^) of respirable silica** |  |  | Low, *n* = 43 |  |  |  |  |  |  | High, *n* = 15 |  |  |  |
| Systolic blood pressure (mm Hg) | 41 | 1.20 | -2.67 7.00 | -20.50 | 21.33 | 0.41 |  | 14 | 3.51 | 1.33 7.00 | -7.67 | 10.00 | 0.02 |
| Diastolic blood pressure (mm Hg) | 41 | 0.87 | -4.83 7.00 | -16.33 | 21.00 | 0.51 |  | 14 | 1.67 | -3.33 5.00 | -8.67 | 12.67 | 0.34 |
| C-reactive protein (mg/L) | 41 | -0.63 | -0.62 0.38 | -13.19 | 3.00 | 0.13 |  | 14 | -0.88 | -1.35 0.00 | -3.34 | 1.19 | 0.04 |
| Homocystein (µmol/L) | 40 | -0.15 | -1.00 1.50 | -10.00 | 8.00 | 0.78 |  | 14 | -0.21 | -1.00 1.00 | -5.00 | 4.00 | 0.77 |
| Cholesterol (mmol/L) | 41 | -0.07 | -0.50 0.30 | -1.60 | 1.60 | 0.50 |  | 14 | -0.26 | -0.70 0.10 | -1.20 | 0.50 | 0.08 |
| High density lipoprotein (mmol/L) | 40 | -0.03 | -0.15 0.10 | -0.50 | 0.40 | 0.41 |  | 14 | -0.03 | -0.10 0.10 | -0.20 | 0.21 | 0.46 |
| Low density lipoprotein (mmol/L) | 41 | 1.29 | 0.90 2.00 | -8.90 | 3.60 | <0.01 |  | 14 | 1.34 | 0.90 1.70 | 0.00 | 2.70 | <0.01 |
| Triglycerides (mmol/L) | 41 | -0.14 | -0.50 0.30 | -4.90 | 1.40 | 0.37 |  | 14 | -0.09 | -0.40 0.10 | -1.00 | 0.90 | 0.48 |
| Pulse, resting (bpm) | 41 | -3.71 | -8.67 1.33 | -23.67 | 29.33 | 0.03 |  | 14 | 1.86 | -6.67 10.67 | -18.67 | 21.00 | 0.57 |
| Serum Amyloid A (mg/L) | 41 | -1.69 | -0.79 0.43 | -35.65 | 6.70 | 0.12 |  | 14 | -1.88 | -1.59 0.41 | -12.15 | 2.90 | 0.13 |
| Fibrinogen (g/L) | 41 | -0.05 | -0.30 0.20 | -1.50 | 0.80 | 0.55 |  | 13 | -0.06 | -0.50 0.40 | -1.10 | 1.00 | 0.72 |
|  |  |  |  |  |  |  |  |  |  |  |  |  |  |
| **75^th^ percentile (0.462** **mg/m^3^) of respirable dust** |  |  | Low, *n* = 43 |  |  |  |  |  |  | High, *n* = 15 |  |  |  |
| Systolic blood pressure (mm Hg) | 42 | 0.89 | -3.83 6.67 | -20.50 | 21.33 | 0.53 |  | 13 | 4.71 | 3.67 7.33 | -7.67 | 12.17 | <0.01 |
| Diastolic blood pressure (mm Hg) | 42 | 0.44 | -5.00 6.00 | -16.33 | 21.00 | 0.73 |  | 13 | 3.10 | -1.67 8.67 | -8.67 | 12.67 | 0.10 |
| C-reactive protein (mg/L) | 42 | -0.58 | -0.75 0.38 | -13.19 | 3.00 | 0.13 |  | 13 | -1.05 | -2.70 0.20 | -4.72 | 1.19 | 0.06 |
| Homocystein (µmol/L) | 41 | -0.34 | -1.00 1.00 | -10.00 | 8.00 | 0.50 |  | 13 | 0.38 | -1.00 3.00 | -5.00 | 4.00 | 0.67 |
| Cholesterol (mmol/L) | 42 | -0.15 | -0.60 0.20 | -1.60 | 1.10 | 0.12 |  | 13 | -0.02 | -0.40 0.20 | -1.00 | 1.60 | 0.93 |
| High density lipoprotein (mmol/L) | 41 | -0.04 | -0.10 0.10 | -0.50 | 0.40 | 0.32 |  | 13 | -0.01 | -0.10 0.10 | -0.20 | 0.21 | 0.84 |
| Low density lipoprotein (mmol/L) | 42 | 1.20 | 0.90 1.91 | -8.90 | 3.20 | <0.01 |  | 13 | 1.64 | 1.10 1.70 | 0.30 | 3.60 | <0.01 |
| Triglycerides (mmol/L) | 42 | -0.16 | -0.50 0.30 | -4.90 | 1.40 | 0.28 |  | 13 | -0.02 | -0.40 0.10 | -1.00 | 1.10 | 0.89 |
| Pulse, resting (bpm) | 42 | -4.65 | -10.33 1.00 | -23.67 | 29.33 | 0.01 |  | 13 | 5.33 | -2.00 10.67 | -11.67 | 21.00 | 0.06 |
| Serum Amyloid A (mg/L) | 42 | -1.46 | -0.88 0.32 | -35.65 | 6.70 | 0.15 |  | 13 | -2.65 | -1.59 0.62 | -13.78 | 2.90 | 0.12 |
| Fibrinogen (g/L) | 42 | -0.05 | -0.40 0.20 | -1.30 | 0.80 | 0.52 |  | 12 | -0.07 | -0.40 0.55 | -1.50 | 1.00 | 0.76 |
|  |  |  |  |  |  |  |  |  |  |  |  |  |  |
| **75^th^ percentile (0.984 mg/m^3^) of dust of PM 0.1-10** |  |  | Low, *n* = 33 |  |  |  |  |  |  | High, *n* =13 |  |  |  |
| Systolic blood pressure (mm Hg) | 31 | 0.68 | -5.33 7.83 | -20.50 | 20.83 | 0.69 |  | 12 | 3.88 | 2.42 5.42 | -0.50 | 7.33 | <0.01 |
| Diastolic blood pressure (mm Hg) | 31 | -0.05 | -5.00 7.00 | -16.33 | 19.00 | 0.97 |  | 12 | 1.97 | -3.33 6.83 | -8.67 | 10.33 | 0.29 |
| C-reactive protein (mg/L) | 31 | -0.90 | -0.90 0.22 | -13.19 | 1.60 | 0.07 |  | 12 | -1.37 | -2.90 -0.18 | -4.72 | 1.19 | 0.02 |
| Homocystein (µmol/L) ^c^ | 31 | -0.58 | -2.00 1.00 | -10.00 | 8.00 | 0.39 |  | 11 | 0.09 | -1.00 3.00 | -5.00 | 4.00 | 0.92 |
| Cholesterol (mmol/L) | 31 | -0.14 | -0.60 0.30 | -0.90 | 1.10 | 0.16 |  | 12 | 0.01 | -0.60 0.45 | -1.20 | 1.60 | 0.97 |
| High density lipoprotein (mmol/L) | 31 | -0.03 | -0.10 0.10 | -0.50 | 0.40 | 0.56 |  | 11 | 0.00 | -0.10 0.10 | -0.20 | 0.21 | 0.98 |
| Low density lipoprotein (mmol/L) | 31 | 1.47 | 1.00 1.90 | -0.10 | 3.20 | <0.01 |  | 12 | 0.59 | 0.55 1.70 | -8.90 | 3.60 | 0.53 |
| Triglycerides (mmol/L) | 31 | -0.07 | -0.50 0.30 | -1.00 | 1.40 | 0.55 |  | 12 | -0.37 | -0.35 0.10 | -4.90 | 1.10 | 0.41 |
| Pulse, resting (bpm) | 31 | -5.08 | -10.33 1.33 | -23.67 | 29.33 | 0.01 |  | 12 | 0.69 | -11.50 10.67 | -18.67 | 21.00 | 0.86 |
| Serum Amyloid A (mg/L) | 31 | -2.27 | -1.21 0.26 | -35.65 | 6.70 | 0.09 |  | 12 | -3.41 | -7.15 0.02 | -13.78 | 2.90 | 0.06 |
| Fibrinogen (g/L) | 30 | -0.13 | -0.40 0.20 | -1.30 | 0.80 | 0.18 |  | 12 | -0.09 | -0.50 0.55 | -1.50 | 1.00 | 0.68 |

**^a^** Inter-quartile range
**^b^** Paired t-test

**Table S6c** Sensitivity analyses including study participants not having worked prior to measurements after vacation: changes in blood pressure, pulse, and biomarkers in low- and high-exposed groups. The groups are dichotomized based on exposure measurements’ 75^th^ percentile concentration levels of respirable silica (0.026 mg/m^3^), respirable dust (0.462 mg/m^3^) and dust of PM 0.1-10 (0.984 mg/m^3^), respectively. A positive value indicates higher point estimates during work than after vacation.

| **Not worked** | ***n*** | **Mean of change** | **IQR ^a^** | **Min** | **Max** | **p-value ^b^** |  | ***n*** | **Mean of change** | **IQR ^a^** | **Min** | **Max** | **p-value ^b^** |
| --- | --- | --- | --- | --- | --- | --- | --- | --- | --- | --- | --- | --- | --- |
| **75^th^ percentile (0.026 mg/m^3^) of respirable silica** |  |  | Low, *n* = 39 |  |  |  |  |  |  | High, *n* = 14 |  |  |  |
| Systolic blood pressure (mm Hg) | 37 | -1.52 | -6.67 3.50 | -20.50 | 20.83 | 0.49 |  | 13 | 4.96 | 4.00 7.33 | -7.67 | 12.17 | 0.02 |
| Diastolic blood pressure (mm Hg) | 37 | -1.46 | -5.67 4.00 | -16.33 | 19.00 | 0.40 |  | 13 | 4.08 | -1.67 9.67 | -8.67 | 15.33 | 0.14 |
| C-reactive protein (mg/L) | 37 | -0.60 | -1.10 0.22 | -13.19 | 7.50 | 0.17 |  | 13 | -1.05 | -2.07 0.20 | -4.72 | 1.19 | 0.06 |
| Homocystein (µmol/L) | 37 | -0.43 | -1.00 1.00 | -10.00 | 6.00 | 0.73 |  | 13 | 0.00 | -1.00 3.00 | -5.00 | 4.00 | 0.36 |
| Cholesterol (mmol/L) | 37 | -0.22 | -0.60 0.20 | -1.60 | 0.70 | 0.12 |  | 13 | -0.04 | -0.40 0.20 | -1.00 | 1.60 | 0.12 |
| High density lipoprotein (mmol/L) | 37 | -0.06 | -0.20 0.10 | -0.50 | 0.30 | 0.15 |  | 13 | 0.01 | -0.01 0.10 | -0.20 | 0.21 | 0.89 |
| Low density lipoprotein (mmol/L) | 37 | 1.47 | 0.90 2.00 | -0.10 | 3.20 | <0.01 |  | 13 | 1.64 | 1.10 1.70 | 0.30 | 3.60 | <0.01 |
| Triglycerides (mmol/L) | 37 | 0.03 | -0.50 0.40 | -1.40 | 1.40 | 0.56 |  | 13 | -0.07 | -0.40 0.00 | -1.00 | 1.10 | 0.23 |
| Pulse, resting (bpm) | 37 | 4.29 | -10.33 1.33 | -23.67 | 29.33 | 0.06 |  | 13 | 4.05 | -2.33 8.33 | -11.67 | 21.00 | 0.48 |
| Serum Amyloid A (mg/L) | 37 | -1.67 | -1.21 0.12 | -35.65 | 10.71 | 0.11 |  | 13 | -2.56 | -1.59 0.62 | -13.78 | 2.90 | 0.20 |
| Fibrinogen (g/L) | 37 | -0.01 | -0.40 0.20 | -1.30 | 1.00 | 0.76 |  | 12 | -0.07 | -0.40 0.55 | -1.50 | 1.00 | 0.89 |
|  |  |  |  |  |  |  |  |  |  |  |  |  |  |
| **75^th^ percentile (0.462** **mg/m^3^) of respirable dust** |  |  | Low, *n* = 38 |  |  |  |  |  |  | High, *n* = 15 |  |  |  |
| Systolic blood pressure (mm Hg) | 37 | 0.31 | -5.25 6.25 | -20.50 | 21.33 | 0.32 |  | 13 | 4.87 | 3.67 7.33 | -7.67 | 12.17 | <0.01 |
| Diastolic blood pressure (mm Hg) | 37 | -0.25 | -5.00 5.50 | -16.33 | 21.00 | 0.27 |  | 13 | 3.98 | -1.67 9.67 | -8.67 | 15.33 | 0.06 |
| C-reactive protein (mg/L) | 37 | -0.39 | -0.90 0.33 | -13.19 | 7.50 | 0.20 |  | 13 | -0.99 | -2.70 0.20 | -4.72 | 1.19 | 0.06 |
| Homocystein (µmol/L) | 37 | -0.23 | -1.00 1.00 | -10.00 | 8.00 | 0.40 |  | 13 | 0.21 | -1.00 3.00 | -5.00 | 4.00 | 1.00 |
| Cholesterol (mmol/L) | 37 | -0.12 | -0.55 0.25 | -1.60 | 1.10 | 0.02 |  | 13 | -0.03 | -0.40 0.20 | -1.00 | 1.60 | 0.84 |
| High density lipoprotein (mmol/L) | 37 | -0.03 | -0.10 0.10 | -0.50 | 0.40 | 0.12 |  | 13 | -0.01 | -0.10 0.10 | -0.20 | 0.21 | 0.82 |
| Low density lipoprotein (mmol/L) | 37 | 1.21 | 0.85 1.96 | -8.90 | 3.20 | <0.01 |  | 13 | 1.54 | 0.90 1.70 | 0.30 | 3.60 | <0.01 |
| Triglycerides (mmol/L) | 37 | -0.15 | -0.50 0.30 | -4.90 | 1.40 | 0.75 |  | 13 | -0.02 | -0.40 0.10 | -1.00 | 1.10 | 0.66 |
| Pulse, resting (bpm) | 37 | -4.02 | -10.33 1.33 | -23.67 | 29.33 | 0.02 |  | 13 | 4.69 | -2.33 10.67 | -11.67 | 21.00 | 0.14 |
| Serum Amyloid A (mg/L) | 37 | -1.15 | -0.99 0.38 | -35.65 | 10.71 | 0.15 |  | 13 | -2.48 | -1.59 0.62 | -13.78 | 2.90 | 0.13 |
| Fibrinogen (g/L) | 37 | -0.01 | -0.35 0.25 | -1.30 | 1.00 | 0.87 |  | 12 | -0.08 | -0.30 0.40 | -1.50 | 1.00 | 0.76 |
|  |  |  |  |  |  |  |  |  |  |  |  |  |  |
| **75^th^ percentile (0.984 mg/m^3^) of dust of PM 0.1-10** |  |  | Low, *n* = 32 |  |  |  |  |  |  | High, *n* =10 |  |  |  |
| Systolic blood pressure (mm Hg) | 30 | -1.19 | -8.00 4.33 | -20.50 | 20.83 | 0.52 |  | 9 | 3.88 | 2.42 5.42 | -0.50 | 7.33 | <0.01 |
| Diastolic blood pressure (mm Hg) | 30 | -1.19 | -6.67 7.00 | -16.33 | 19.00 | 0.46 |  | 9 | 1.97 | -3.33 6.83 | -8.67 | 10.33 | 0.13 |
| C-reactive protein (mg/L) | 30 | -0.99 | -1.11 0.20 | -13.19 | 1.60 | 0.05 |  | 9 | -1.37 | -2.90 -0.18 | -4.72 | 1.19 | 0.05 |
| Homocystein (µmol/L) ^c^ | 30 | -0.63 | -1.00 1.00 | -10.00 | 6.00 | 0.32 |  | 9 | 0.09 | -1.00 3.00 | -5.00 | 4.00 | 0.76 |
| Cholesterol (mmol/L) | 30 | -0.17 | -0.60 0.20 | -0.90 | 0.70 | 0.07 |  | 9 | 0.01 | -0.60 0.45 | -1.20 | 1.60 | 0.97 |
| High density lipoprotein (mmol/L) | 30 | -0.05 | -0.10 0.10 | -0.50 | 0.30 | 0.23 |  | 9 | 0.00 | -0.10 0.10 | -0.20 | 0.21 | 0.43 |
| Low density lipoprotein (mmol/L) | 30 | 1.49 | 0.90 2.00 | -0.10 | 3.20 | <0.01 |  | 9 | 0.59 | 0.55 1.70 | -8.90 | 3.60 | <0.01 |
| Triglycerides (mmol/L) | 30 | 0.05 | -0.50 0.60 | -1.00 | 1.40 | 0.66 |  | 9 | -0.37 | -0.35 0.10 | -4.90 | 1.10 | 0.89 |
| Pulse, resting (bpm) | 30 | -5.38 | -12.67 1.33 | -23.67 | 29.33 | 0.01 |  | 9 | 0.69 | -11.50 10.67 | -18.67 | 21.00 | 0.50 |
| Serum Amyloid A (mg/L) | 30 | -2.49 | -2.11 -0.06 | -35.65 | 6.70 | 0.07 |  | 9 | -3.41 | -7.15 0.02 | -13.78 | 2.90 | 0.10 |
| Fibrinogen (g/L) | 29 | -0.11 | -0.40 0.10 | -1.30 | 0.80 | 0.23 |  | 9 | -0.09 | -0.50 0.55 | -1.50 | 1.00 | 0.85 |

**^a^** Inter-quartile range
**^b^** Paired t-test

**Table S7** Effect estimates expressed as β-values with 95 % confidence interval (CI) of blood pressure, pulse and biomarkers in study participants associated with years of work in dust-exposed jobs, adjusted for BMI, and BMI and age (years), respectively. Linear regression with continuous variables. The β represents the change in each variable with one year of work in dust-exposed jobs.

|  | ***Working years*** |  |  |  |  |  |  |  |  |
| --- | --- | --- | --- | --- | --- | --- | --- | --- | --- |
|  | Crude β | 95 % CI |  | Adj β  (BMI ^a^) | 95 % CI |  | Adj β  (BMI ^a^, age) | 95 % CI |  |
| Systolic blood pressure (mm Hg) | 0.16 | -0.13, 0.45 |  | 0.10 | -0.17, 0.37 |  | -0.03 | -0.40, 0.34 |  |
| Diastolic blood pressure (mm Hg) | 0.19 | -0.05, 0.43 |  | 0.14 | -0.08, 0.37 |  | -0.12 | -0.42, 0.17 |  |
| C-reactive protein (mg/L) | 0.00 | -0.06, 0.06 |  | -0.01 | -0.06, 0.05 |  | -0.03 | -0.10, 0.05 |  |
| Homocysteine (µmol/L) | 0.06 | -0.01, 0.13 |  | 0.07 | -0.00, 0.14 |  | 0.11 | 0.02, 0.21 |  |
| Cholesterol (mmol/L) | 0.03 | 0.01, 0.05 |  | 0.03 | 0.01, 0.05 |  | 0.01 | -0.01, 0.04 |  |
| High density lipoprotein (mmol/L) | 0.00 | -0.00, 0.01 |  | 0.00 | -0.00, 0.01 |  | 0.00 | -0.01, 0.01 |  |
| Low density lipoprotein (mmol/L) | 0.03 | 0.01, 0.04 |  | 0.03 | 0.02, 0.05 |  | 0.03 | 0.01, 0.05 |  |
| Triglycerides (mmol/L) | -0.00 | -0.03, 0.03 |  | 0.00 | -0.03, 0.02 |  | -0.03 | -0.06, 0.01 |  |
| Pulse, resting (bpm) | 0.06 | -0.15, 0.28 |  | 0.03 | -0.18, 0.23 |  | -0.08 | -0.36, 0.20 |  |
| SerumAmyloid A (mg/L) | 0.05 | -0.01, 0.12 |  | 0.04 | -0.02, 0.10 |  | 0.03 | -0.05, 0.11 |  |
| Fibrinogen (g/L) | 0.01 | -0.00, 0.02 |  | 0.01 | -0.01, 0.02 |  | -0.00 | -0.02, 0.01 |  |

^a^ Body mass index
